# A baseline sarcopenia index based on creatinine/cystatin C for the prediction of stroke recurrence and mortality in older survivors of first ischemic strokes

**DOI:** 10.3389/fpubh.2023.1049738

**Published:** 2023-09-28

**Authors:** Lisha Hou, Xiaoyan Chen, Cairong Zhu

**Affiliations:** ^1^West China School of Public Health and West China Fourth Hospital, Sichuan University, Chengdu, China; ^2^National Clinical Research Center for Geriatrics, West China Hospital, Sichuan University, Chengdu, Sichuan Province, China; ^3^Zigong Psychiatric Research Center, Zigong Affiliated Hospital of Southwest Medical University, Zigong, Sichuan Province, China

**Keywords:** ischemic stroke, sarcopenia index, creatinine, cystatin C, recurrence, mortality

## Abstract

**Objective:**

Older adults individuals have a higher risk of stroke recurrence, leading to high mortality and disability rates, which, in turn, hinders the achievement of healthy aging. This study aimed to assess the utility of a baseline sarcopenia index (SI) based on serum creatinine (Cr)/cystatin C (CysC) as a prognostic marker for the risk of stroke recurrence and mortality in first-ever ischemic stroke older survivors (ISOS).

**Materials and methods:**

Data were obtained from an ischemic stroke cohort study. The baseline information was collected from medical records and face-to-face interviews with patients admitted between January 2010 and June 2016. Follow-up information was obtained from telephone interviews every 3 months to determine stroke recurrence and survival status. The SI was calculated from the Cr and CysC values in the medical records as Cr/CysC × 100. Using the first quantile of the SI as the cut-off value, the study participants were divided into the low muscle-mass group (low SI) and the high muscle-mass group (high SI). Cox regression analysis was used to assess the association between SI and recurrence and mortality.

**Results:**

A total of 415 first-ever ISOS were enrolled, including 242 (58.31%) male and 173 (41.69%) female participants. In the high-SI group, the relapse and mortality rates were lower than those in the low-SI group (relapse: 20.58% vs. 30.77%; mortality:13.5% vs. 29.81%). After adjusting for confounding factors, the high-SI group was found to have a lower risk of relapse and mortality than the low-SI group (relapse: HR = 0.571; mortality: HR = 0.294).

**Conclusion:**

The SI was predictive of the long-term prognosis of IS recurrence and mortality in first-ever ISOS. After discharge, in addition to conventional medication, it is recommended that patients with low SI values actively receive treatment for sarcopenia to reduce the risk of stroke recurrence and mortality and promote healthy aging.

## Introduction

In May 1987, the World Health Organization Assembly first proposed the term “healthy aging.” Healthy aging has two levels of meaning, one of which indicates a healthy old age with disability or loss of function only apparent in later years and of short duration, resulting in improved quality of life in the older adults and a more meaningful later life. Disability or loss of function in the older adults is often associated with stroke. Stroke has a high likelihood of recurrence and is associated with high rates of both disability and mortality (1–3).

Ischemic stroke (IS) is a common type of stroke. It is estimated that approximately 17 million people throughout the world suffer a first IS. The recurrence rate of IS ranges from 16 to 29% in the United States (4) and has been found to be 29.43% in China, where it is the leading cause of death and adult disability (5). The high rate of recurrence is directly proportional to increased mortality (6, 7) as well as a reduced capacity to compensate for functional injury (6). Therefore, the prediction of stroke prognosis is extremely important for its overall prevention and treatment.

Previous studies have shown that older patients have an elevated risk of stroke recurrence (8–10) and patients with sarcopenia before stroke have poor functional outcomes, including longer hospital stays (11), worse neurological impairment (11, 12), and reduced ability to walk (13). Sarcopenia is a common disease in the older population and is associated with significant risks of impaired mobility, falls, reduced quality of life, and mortality (14). Sarcopenia is generally diagnosed by the presence of reduced skeletal muscle mass and low muscle function (15). The assessment of muscle mass is commonly made by computed tomography (CT), magnetic resonance imaging (MRI), dual-energy X-ray absorptiometry (DXA), and bioelectrical impedance analysis (BIA). However, these methods have disadvantages, as CT and MRI have no clear cut-offs for muscle mass, DXA is time-consuming, and BIA requires patients to maintain a specific position for extended periods, often difficult for stroke patients. While muscle function can also be assessed by handgrip strength and gait speed, this can be challenging for patients with limb hemiplegia.

It would thus be economical and effective to evaluate sarcopenia in stroke patients using routine detection indices. Several recent studies (16–18) have shown that the ratio of creatinine to cystatin C can be used as an indicator of both muscle mass and muscle function and, therefore, the Creatinine/CystatinC (Cr/CysC)*100 was proposed as a sarcopenia index (SI). A three-month follow-up study (19) demonstrated that the Cr/CysC ratio at admission was an effective predictor of 30-day mortality and poor long-term prognosis (modified Rankin Scale score ≥ 4) in patients with acute IS. However, the authors found no association between the Cr/CysC ratio and stroke recurrence. Wang et al. (20) reported that the serum Cr/CysC ratio at admission could be used to predict poor long-term prognosis in neurocritically ill cases (including patients with IS and patients without IS) but not 30-day mortality. The inconsistencies between these findings may have resulted from differences in follow-up times or subjects. There is a lack of long-term follow-up investigations on the relationship between SI and IS prognosis (recurrence or death). The present study was based on a long-term cohort of IS survivors and explored the relationship between SI and recurrence and mortality in first-ever ISOS, with the aim of providing a reference for the prevention of stroke recurrence and the promotion of healthy aging.

## Materials and methods

### Study population

**Figure 1:**
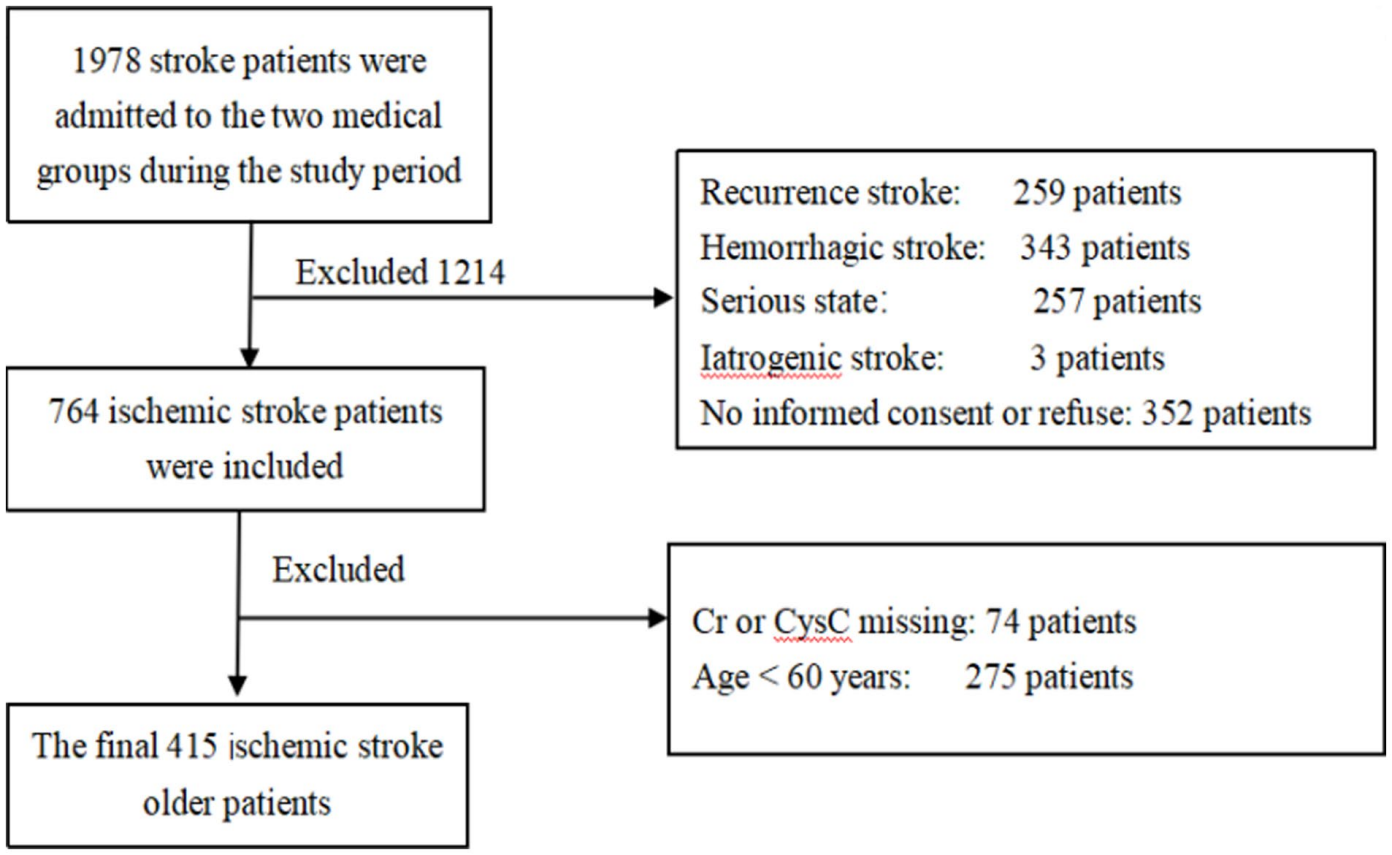
Flow chart of study participants.

The dataset was derived from an IS cohort study. Details of the cohort design were reported previously (21). Between January 2010 and June 2016, a total of 764 patients with a primary and first diagnosis of IS were recruited during admission to two medical groups in the Department of Neurology, West China Hospital, Sichuan University. From this cohort, we chose patients who were 60 years old and over at admission. Patients with iatrogenic stroke, such as carotid endarterectomy, cardiac surgery, or angioplasty, were excluded due to medical issues and compliance. Patients with eGFR <15 mL/min/1.73 m^2^, those who had received renal replacement treatment, had acute kidney injury, or refused clinical follow-up, were also excluded. The inclusion criteria are shown in the flow chart (Figure 1). The study was approved by the Ethics Committee of the West China Hospital, Sichuan University, Chengdu, China. All patients signed informed consent.

### Study design

After obtaining written consent, trained interviewers used a semi-structured questionnaire in a face-to-face setting to obtain information on the basic characteristics and lifestyle of patients before the stroke. Proxies were interviewed when patients had cognitive or language impairments. At baseline, the researchers collected basic information including age, sex, marital status, education, physiological and biochemical indices, TOAST classification, lesion location, smoking history, drinking history, BMI, Rankin score, chronic disease, and exercise from the medical records and face-to-face interviews. During the follow-up, the researchers conducted phone interviews every 3 months after discharge until June 2016 to obtain information, including stroke recurrence (the first relapse was selected as the terminal event) and death (all-cause mortality). According to the purpose of this study, we retrospectively collected further data, including cystatin C and ALB levels, from the patient’s medical records.

### Sarcopenia index

We used the following equation to calculate the SI: serum Cr/CysC × 100. Based on a previous study (22), we divided all participants into two groups using the first quantile as the cut-off value, namely, the low muscle-mass group, representing the lowest to the first quartile and high muscle-mass group, above the first quartile.

### Statistical analysis

All data were analyzed with SAS 9.4 software (Statistical Analysis System, SAS Institute Inc., Cary, NC, United States); reference.^1^ All quantitative data are presented as mean and standard deviation or median and interquartile based on distribution. Categorical variables are presented as frequencies and proportions. Various tests, including the rank-sum, Pearson’s chi-square, and Student’s *t*-test, were used for the comparison of the baseline features. The Kaplan–Meier method was used to evaluate survival outcomes to recurrence and death in relation to SI among different patients. The correlations between SI and stroke recurrence and mortality were determined using Cox regression analysis. A value of *p* < 0.05 indicated statistical significance.

## Results

### General characteristics

A total of 415 older first-ever IS patients were recruited; these included 242 (58.31%) males and 173 (41.69%) females. The ages of the subjects ranged from 60 to 90 years at the baseline. The median follow-up time was 42.75 months. Table 1 presents the characteristics of the different groups in relation to recurrence and death status. Compared with patients with high SI, those with low SI were more likely to relapse and die (*p* < 0.05).

**Table 1:** Baseline characteristics of participants by the recurrence and death.

Variable	Recurrence			Death		
	No (*n* = 319)	Yes (*n* = 96)	*P*	No (*n* = 342)	Yes (*n* = 73)	*P*
Age, years, median(IQR)	69(64,75)	70(64,77.75)	0.214	68(63,74)	74(68.5,79.5)	<0.001
Sex *n*(%)			0.448			0.947
Male	190(78.19)	53(21.81)		200(82.3)	43(17.7)	
Female	129(75)	43(25)		142(82.56)	30(17.44)	
Marital status *n*(%)			0.59			0.025
Married	49(74.24)	17(25.76)		48(72.73)	18(27.27)	
Single/divorced, widowed	269(77.3)	79(22.7)		293(84.2)	55(15.8)	
Education *n*(%)			0.09			0.052
Elementary school and below	134(72.83)	50(27.17)		144(78.26)	40(21.74)	
Junior high school and above	183(79.91)	46(20.09)		196(85.59)	33(14.41)	
Toast classification *n*(%)			0.884			0.635
LAA	150(77.32)	44(22.68)		158(81.44)	36(18.56)	
SAOL	118(75.64)	38(24.36)		132(84.62)	24(15.38)	
CE	51(78.46)	14(21.54)		52(80)	13(20)	
Lesion location *n*(%)			0.077			0.741
Right	92(77.97)	26(22.03)		98(83.05)	20(16.95)	
Left	88(81.48)	20(18.52)		86(79.63)	22(20.37)	
Both	52(82.54)	11(17.46)		51(80.95)	12(19.05)	
Others	87(69.05)	39(30.95)		107(82.54)	19(17.46)	
Smoking history *n*(%)			0.208			0.379
No	153(74.27)	53(25.73)		166(80.58)	40(19.42)	
Yes	163(79.51)	42(20.49)		172(83.9)	33(16.1)	
Drinking history *n*(%)			0.744			0.094
No	217(76.41)	67(23.59)		228(80.28)	56(19.72)	
Yes	102(77.86)	29(22.14)		114(85.07)	17(14.93)	
BMI, kg/m^2^, mean(SD)	23.26(5.11)	23.29(6.46)	0.971	23.46(5.32)	22.38(5.95)	0.126
eGFR, ml/min/1.73 m^2^, mean(SD)	84.04(23.85)	83.63(23.48)	0.882	84.93(23.98)	79.34(22.13)	0.068
ALB, g/L, median(iqr range)	41.25(38.55,43.6)	41.1(38.2,43.5)	0.845	41.5(38.9,43.8)	40.3(37.25,42.1)	0.001
Rank score *n*(%)			0.547			0.008
0–2	96(78.69)	26(21.31)		110(90.16)	12(9.84)	
3–5	221(75.95)	70(24.05)		231(79.38)	60(20.62)	
Hypertension *n*(%)			0.427			0.139
No	96(74.42)	33(25.58)		101(78.29)	28(21.71)	
Yes	223(77.97)	63(22.03)		241(84.27)	45(15.73)	
Diabetes *n*(%)			0.728			0.853
No	220(76.39)	68(23.61)		238(82.64)	50(17.36)	
Yes	99(77.95)	28(22.05)		104(81.89)	23(18.11)	
Hyperlipidemia *n*(%)			0.964			0.184
No	209(77.12)	62(22.88)		219(80.81)	52(19.19)	
Yes	110(76.92)	33(23.08)		123(86.01)	20(13.99)	
Heart disease *n*(%)			0.32			0.663
No	242(78.06)	68(21.94)		254(81.94)	56(18.06)	
Yes	77(73.33)	28(26.67)		88(83.81)	17(16.19)	
Peripheral vascular disease *n*(%)			0.861			0.905
No	306(76.88)	92(23.12)		328(82.42)	70(17.58)	
Yes	12(75)	4(25)		13(81.25)	3(18.75)	
Medication compliance *n*(%)			0.004			0.361
Good	143(83.14)	29(16.86)		148(86.05)	24(13.95)	
Bad	137(70.26)	58(29.74)		161(82.56)	34(17.44)	
Healthy exercise *n*(%)			0.837			0.505
No	160(77.29)	47(22.71)		168(81.16)	39(18.84)	
Yes	159(76.44)	49(23.56)		174(83.65)	34(16.35)	
SI. *n*(%)			0.033			<0.001
Low SI	72(69.23)	32(30.77)		73(70.19)	31(29.81)	
High SI	247(79.42)	64(20.58)		269(86.5)	42(13.5)	

In this study, the first quartile of the sarcopenia index was 76.12, and subjects were divided into two groups, namely the low-SI (<76.12) and high-SI groups (≥76.12). We demonstrated differences in age, sex, marital status, drinking history, smoking history, and eGFR between the low and high muscle-mass groups (Table 2).

**Table 2:** Baseline characteristics of participants according to the sarcopenia index (SI).

Variable	Low SI (*n* = 104)	High SI (*n* = 311)	*p*
Age, years, Median (iqr range)	72(65.5–79)	69(64–74)	<0.01
Sex *n*(%)			<0.01
Male	39(37.50)	203(65.27)	
Female	65(62.50)	108(34.73)	
Marital status *n*(%)			0.019
Married	24(23.30)	42(13.50)	
Single/divorced, widowed	79(76.70)	269(86.50)	
Education *n*(%)			0.202
Elementary school and below	51(50.00)	133(42.77)	
Junior high school and above	51(50.00)	178(57.23)	
Toast classification *n*(%)			0.7
LAA	47(45.19)	147(47.27)	
SAOL	38(36.54)	118(37.04)	
CE	19(18.27)	46(14.79)	
Lesion location *n*(%)			0.485
Right	33(31.73)	85(27.33)	
Left	30(28.85)	78(25.08)	
Both	12(11.54)	51(16.40)	
Others	29(27.88)	97(31.19)	
Smoking history *n*(%)			0.01
No	63(61.17)	143(46.43)	
Yes	40(38.83)	165(53.57)	
Drinking history *n*(%)			0.008
No	82(78.85)	202(64.95)	
Yes	22(21.15)	109(35.05)	
BMI, kg/m^2^, mean(SD)	22.90(3.70)	23.64(4.11)	0.105
eGFR, ml/min/1.73 m^2^, mean(SD)	95.30(28.66)	80.14(20.53)	<0.01
ALB, g/L, median(iqr range)	40.15(37.6–42.50)	41.65(39.10–43.80)	0.624
Rank score *n*(%)			0.209
0–2	25(25.00)	97(31.51)	
3–5	78(75.00)	213(68.49)	
Hypertension *n*(%)			0.165
No	38(36.54)	91(29.26)	
Yes	66(63.46)	220(70.74)	
Diabetes *n*(%)			0.093
No	79(75.96)	209(67.20)	
Yes	25(24.04)	102(32.80)	
Hyperlipidemia *n*(%)			0.241
No	73(70.19)	198(63.87)	
Yes	31(29.81)	112(36.13)	
Heart disease *n*(%)			0.081
No	71(68.27)	239(76.85)	
Yes	33(31.73)	72(23.15)	
Peripheral vascular disease *n*(%)			0.564
No	99(95.19)	299(96.45)	
Yes	5(4.81)	11(3.55)	
Medication compliance *n*(%)			0.480
Good	47(50.00)	125(45.79)	
Bad	47(50.00)	148(54.21)	
Healthy exercise *n*(%)			0.515
No	49(47.12)	158(50.80)	
Yes	55(52.88)	153(49.20)	

LAA, large-artery atherosclerosis; SAOL, small-artery occlusion lacunnar; CE, Cardioembolism; ALB, Serum albumin.

### Recurrence

**Figure 2:**
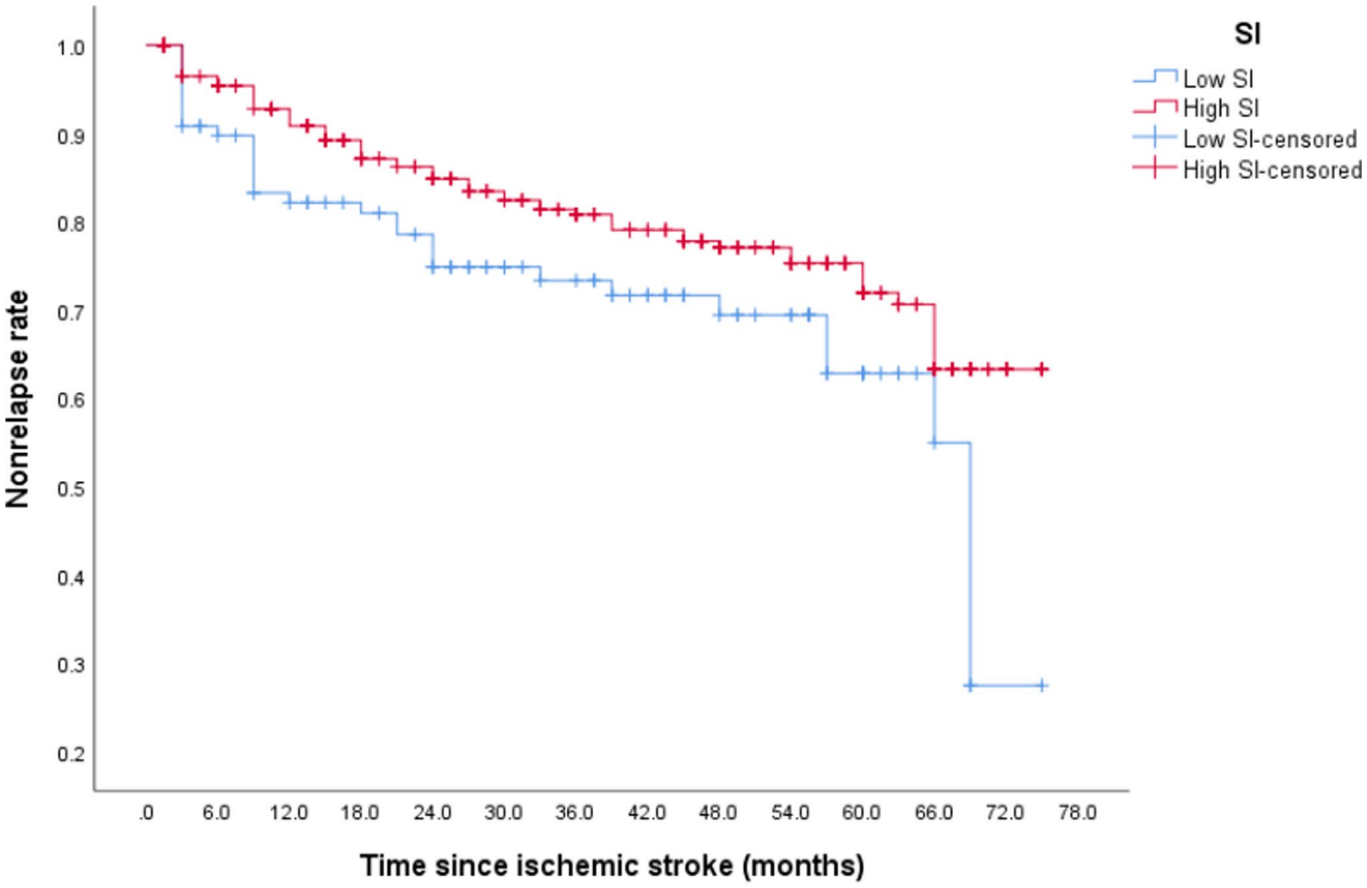
The survival curve of recurrence was evaluated by sarcopenia index (SI).

After discharge from the hospital, 96 (23.13%) older patients with their first IS experienced stroke recurrence, which occurred within 1 year of discharge in 42 patients. During the follow-up period, 64 patients (30.77%) in the low-SI group experienced recurrence while compared with 32 (20.58%) in the high-SI group. The relapse rate at 75 months was 78.50% in the low-SI group and 38.60% in the high-SI group. The median survival time for recurrence in the low and high muscle-mass groups was 66.55 months and > 75 months, respectively, and this difference between the two groups was statistically significant (Figure 2; *p* = 0.02). Table 3 shows that after adjusting for confounding factors, the high-SI group had a lower relapse risk than the low-SI group (HR = 0.571, 95%CI: 0.336–0.969, *p* = 0.038).

**Table 3:** Correlations between SI and recurrence and mortality.

Variable	High SI vs. Low SI	
	*P* value	HR (95% *CI*)
Recurrence		
Model 1	0.023	0.61(0.399–0.934)
Model 2	0.038	0.571(0.336–0.969)
Mortality		
Model 1	<0.01	0.442(0.277–0.705)
Model 2	<0.01	0.294(0.155–0.558)

Model 1: non-adjusted model. Model 2: adjusting for age, sex, marital status, education, toast classification, lesion location, Smoking history, drinking history, ALB level, eGFR, BMI, rank score, hypertension, diabetes, hyperlipidemia, heart disease, peripheral vascular disease, medication compliance, healthy exercise.

### Mortality

**Figure 3:**
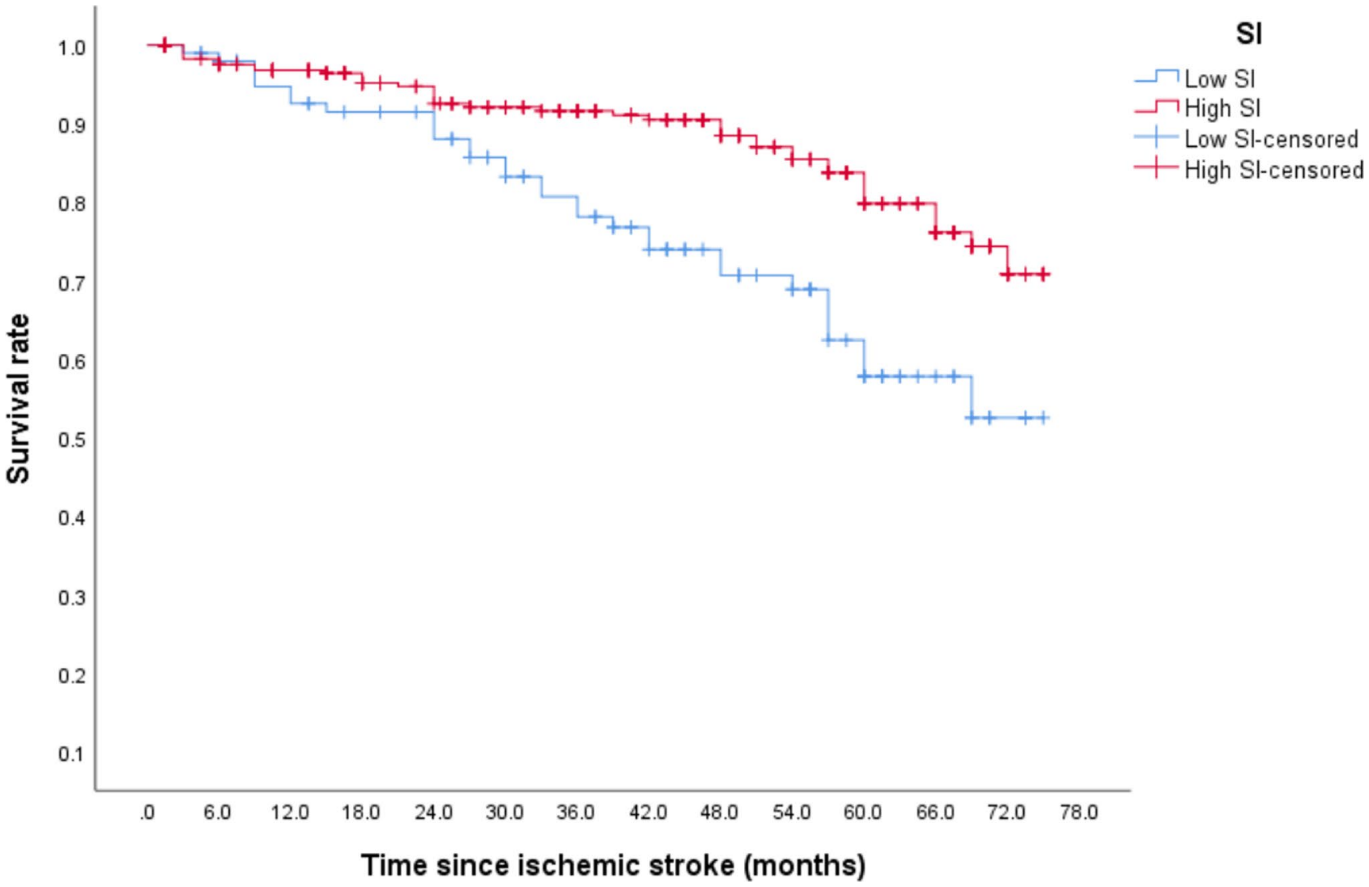
The survival curve of death was evaluated by SI.

After discharge, 73 patients (17.4%) died, with 16 (21.3%) within 1 year of discharge. During the follow-up period, the mortality of the low- and high-SI groups was 29.81 and 13.5%, respectively. Although the median overall survival time in both groups was >75 months, the discrepancy between the two groups was statistically significant (Figure 3; *p* < 0.001). Table 3 illustrates that after adjusting for confounding factors, the high-SI group had a lower mortality risk than the low-SI group (HR = 0.294, 95%CI: 0.155–0.558, *p* < 0.01).

## Discussion

In the present research, 96 (23.13%) older patients with their first IS experienced stroke recurrence, with 72 (17.4%) of patients dying during the median 42.75 months’ follow-up. We found a significant association between SI with both stroke recurrence and mortality among first-ever ISOS. The high-SI group had a lower relapse risk and mortality, suggesting that active intervention to improve SI may prevent stroke recurrence and death and promote healthy aging in the older adults.

First-ever ISOS patients in the low-SI group showed significantly higher mortality. Patients with high SI scores were 0.294 times less likely to die than patients with lower scores, which is similar to the findings of a previous study (19). There are several possible reasons for this. First, the low-SI group represents the low muscle-mass group which includes patients who tend to have low levels of protein involvement, associated with malnutrition. Patients with low muscle mass tend to be malnourished, which affects their prognosis (23). Second, patients with sarcopenia tend to have difficulty swallowing and are more likely to develop aspiration pneumonia (24). Third, sarcopenia patients are more likely to fall (15), causing fractures that can lead to death in older adults. Fourth, this study confirmed that low muscle mass was associated with a higher rate of stroke, also potentially leading to death. Fifth, sarcopenia is closely linked to mechanical ventilation and infectious complications, both of which are frequently fatal in older adults (25, 26). All these demonstrate the important role of the SI level in the prognosis of the first IS.

The findings of the present study showed that patients with high skeletal muscle mass had a lower rate of stroke recurrence. It has also been found that patients with low skeletal muscle mass may be at a higher risk of developing IS (13). This could result from fewer changes in the white matter and silent infarctions that are known to be associated with higher skeletal muscle mass, which may prevent stroke occurrence and thus reduce the recurrence rate (27).

In the present study, we chose the Cr/CysC ratio rather than muscle mass itself to assess the presence or absence of muscle loss. There were several reasons for this choice. Serum creatinine is an endogenous product derived from creatine phosphate and its production is influenced by skeletal muscle. However, serum creatinine levels are also affected by renal function, thus limiting the reliability of this biomarker in assessing muscle (28). Cystatin C is a small protein originating in nucleated cells that is reabsorbed by proximal tubules and completely decomposed. Its production is less affected by muscle mass. Therefore, Cr/CysC can effectively assess muscle mass without renal imaging (18).

The study has some strengths. The availability of data at multiple time points is one of the strengths of this study. Over the median follow-up period of 42.75 months, patients were assessed every 3 months. Therefore, we could compile accurate survival curves for the low-and high-muscle mass groups throughout this period. However, the study does have some limitations. First, we assessed the outcomes every 3 months by phone, making the data prone to memory bias. The rate of stroke recurrence might have been underestimated as some patients may have experienced a transient ischemic attack without recognizing it. Second, we only considered the first relapse of patients but some cases had more than one stroke recurrence. Third, as almost 50% of patients lacked NIHSS scores in their medical records, the modified Rankin Scale (mRS) was included in the model as a confounding factor. Fourth, the SI we used lacks a comparison with the gold standard for diagnosing sarcopenia, so the SI as an indicator of sarcopenia in the stroke population requires further study. Therefore, future research should collect more comprehensive basic information for analysis.

## Conclusion

The study found a significant association between the sarcopenia index and both stroke recurrence and mortality in individuals with their first-ever ischemic stroke. These findings provide important insights and references for the secondary prevention of stroke. It is imperative to prioritize the modification of risk factors that contribute to both sarcopenia and stroke, such as smoking, unhealthy diet, and a lack of physical activity, in conjunction with regular medication after discharge. Furthermore, regular assessment of the sarcopenia index or sarcopenia status in stroke survivors is important. It is also recommended that patients receive personalized nutritional support and rehabilitation exercises to improve muscle mass and functionality. By implementing active and effective interventions and treatments aimed at reducing stroke recurrence, we can successfully prevent the occurrence of secondary strokes and promote healthy aging.

## Data availability statement

The raw data supporting the conclusions of this article will be made available by the authors, without undue reservation.

## Ethics statement

The studies involving humans were approved by the Ethics Committee of the West China Hospital, Sichuan University, Chengdu, China. All patients signed informed consent. The studies were conducted in accordance with the local legislation and institutional requirements. The participants provided their written informed consent to participate in this study.

## Author contributions

LH, XC, and CZ: study concept and design, and analysis and interpretation of data. LH and XC: acquisition of data and drafting of the manuscript. CZ: critical revision of the manuscript for important intellectual content. All authors contributed to the article and approved the submitted version.

## Funding

This research was supported by grants from the National Natural Science Foundation of China (grant nos. 82173618, 81673273, and 30600511).

## Conflict of interest

The authors declare that the research was conducted in the absence of any commercial or financial relationships that could be construed as a potential conflict of interest.

## Publisher’s note

All claims expressed in this article are solely those of the authors and do not necessarily represent those of their affiliated organizations, or those of the publisher, the editors and the reviewers. Any product that may be evaluated in this article, or claim that may be made by its manufacturer, is not guaranteed or endorsed by the publisher.

## Footnotes

^1^ https://www.sas.com/en_us/legal/editorial-guidelines.html
